# Thiol/disulphide homeostasis in intensive care unit patients with sepsis and septic shock

**DOI:** 10.3906/sag-1905-148

**Published:** 2020-06-23

**Authors:** Hamit YILDIZ

**Affiliations:** 1 Department of Internal Medical Sciences and Critical Care, Faculty of Medicine, Gaziantep University, Gaziantep Turkey

**Keywords:** Oxidative stress, antioxidant level, tissue damage, critical care patients

## Abstract

**Background/aim:**

Sepsis is a condition caused by infection followed by unregulated inflammatory response which may lead to organ dysfunction. The aim of this study is to be the first in the literature and it has been designed to show the thiol/disulphide changes in patients with sepsis and septic shock and their correlation with acute phase reactants.

**Material and methods:**

A total of 113 patients (septic shock 53 and sepsis 60) and 60 healthy control subjects have been enrolled in this study from the period February 2018 to 2019. The patients were divided in 2 groups: nonsurvivors (74) and survivors (39). The investigation includes measurements of native thiol, total thiol, dynamic disulphide bond, oxidized thiol ratio, reduced thiol ratio and thiol oxidation reduction ratio, erythrocyte sedimentation rate, C-reactive protein, and procalcitonin.

**Results:**

The findings of this study suggest that changes in thiol levels play a role in the pathogenesis of patients with sepsis and septic shock.

**Conclusions:**

Thiol/disulphide homeostasis is impaired in patients with sepsis and septic shock. Understanding the role of thiol/disulphide homeostasis in sepsis and septic shock may provide different therapeutic intervention strategies for patients.

## 1. Introduction:

Sepsis, an exaggerated response to infection by the body, is a frequent problem of intensive care units that respond to physiological and pathological biochemical changes [1]. Although the actual incidence is unknown, it is known that sepsis is still an important cause of mortality in intensive care units worldwide [2]. Concomitant factors such as uncontrolled diabetes mellitus, organ transplantation, surgical history, cirrhosis, and congestive heart failure may increase patients’ susceptibility to sepsis. It is known that the level of molecules such as reactive species and/or free radicals increase in the pathogenesis of sepsis. 

Oxidative stress is an undesirable condition with adverse consequences for the body. Oxidative stress state is the disruption of the balance between the level of antioxidant and oxidant molecules [3]. Overproduction of reactive oxidative species disrupts the structure of proteins and lipids. Due to this effect, breaks in DNA structures and oxidation in proteins and lipids of cell membrane occur [4,5]. A breakdown in the mitochondrial electron transmission systems and an increase in the inducible nitric oxide synthesis are the most important changes that occur [3,6,7]. These mechanisms in sepsis cause multiple organ failure that lead to myocardial depression, cellular dysfunction, endothelial damage, and vascular catecholamine hyporesponsiveness.

There are lots of commercial tests to evaluate the levels of oxidant and antioxidant molecules in human body. Thiols are antioxidant molecules containing sulfhydryl (-SH) group consisting of hydrogen, sulphide, and carbon atoms. Electrons from reactive oxygen molecules are transferred to thiol in the human body and oxidation of these molecules is made by disulphide bonds. Bonding of disulphide structures is reversible [8]. The amount of this conversion may vary according to the level of oxidative stress. Disruption of thiol-disulphide homeostasis, which develops as a result of the increase of oxidative molecules, should be evaluated as a precursor of possible diseases that may occur in the human body [9].

The aim of this research is to assess and compare the relationship between sepsis and septic shock and thiol-disulphide, which is an important cause of mortality in intensive care units, and to compare them with healthy controls. In this study, it is hypothesized that underlying mechanisms of sepsis affect thiol-disulphide homeostasis in the organism.

## 2. Materials and methods:

### 2.1 Study design and patients

This study has been made with a total of 60 patients (30 female and 30 male) with sepsis, 53 patients (25 female and 28 male) with septic shock according to the European Society of Intensive Care Medicine and the Society of Critical Care Medicine 2016 criteria and 50 healthy volunteers (25 female and 25 male), with their written, informed consent. Approval of the Ethics Committee of Gaziantep University Faculty of Medicine has been obtained for this study. Particular data regarding medical record, age, and sex have been obtained for each participant. In the history of healthy control subjects included in the study, there was no history of malignancy, systemic disease, rheumatic disease, and drug use.

### 2.2 Blood sampling and analytic procedure

Venous blood samples containing 10 mL blood have been collected from patients and control groups. Blood samples have been taken from patients diagnosed with sepsis and septic shock in intensive care unit. Blood samples to measure thiol and disulphide levels were centrifuged for 10 min at 1500 rpm within the first 30 min after collection from subjects. These serum samples have been stored in **–**80 °C until biochemical analysis is made.

### 2.3 Laboratory method

Thiol/disulphide homeostasis tests were measured using a new method, automated spectrophotometric method which is available commercially (Rel Assay Diagnostics, Turkey) [9]. In this method, sodium borohydride was used to reduce reducible disulphide bonds to free functional thiols. NaBH4 formaldehyde was used to reduce unused reducing sodium borohydride. Native thiol level (NTL) and total thiol level (TTL) were determined after the reaction with 5,5′-dithiobis-(2-nitrobenzoic) acid (DTNB) and their levels were measured ultimately. Half of the difference between the results obtained by the subtraction of native thiol (-SH) amount from total thiol (-SH+-S-S-) content indicated the disulphide (-S-S-) level. In addition, thiol oxidation reduction ratio (-SH) × 100/ (-S-S-), oxidized thiol ratio ((-S-S) x 100/ (-SH+-S-S-)), and reduced thiol ratio [-SH × 100/(-SH+-S-S-)] have been calculated using these parameters. 

### 2.4 Statistical analysis

In the present study, compliance of variables such as native thiol, total thiol, dynamic disulphide bond, oxidized thiol ratio, reduced thiol ratio, and thiol oxidation reduction ratio with normal distribution have been evaluated using histograms, variation coefficients, skewness, sharpness, detrended normality graph and Kolmogorov–Smirnov test. Median (minimum/maximum) has been used to present descriptive statistics of variables without normal distribution. Mean standard deviation (SD) values of variables with normal distribution are provided (mean ± SD, 95% CI). ANOVA test has been used to evaluate differences between groups (sepsis, septic shock, and control) in terms of native thiol level (NTL), total thiol level (TTL), dynamic disulphide bond level, reduced thiol ratio (RTR, and oxidized thiol ratio (OTR). Kruskal–Wallis test has been used to compare thiol oxidation reduction ratio (TORR) variable within groups and Mann–Whitney U test was used by making bonferroni correction to compare the 2 groups. The analysis of differences in continuous variables between survivor and non survivor groups have been performed using Mann–Whitney U test, in cases where the data distribution is nonnormal, and student’s t test in cases where the data distribution resembles normal distribution. The associations between continuous variables have been analysed using the nonparametric Spearmen correlation test in cases where the data distribution has not been consistent with normal distribution. The Pearson correlation test has been used for continuous variables that showed a normal distribution. IBM SPSS version 24 (IBM Corp., Armonk NY, USA) and MS-Excel 2010 have been used for statistical analysis and calculations. Statistical significance level has been defined as P < 0.05.

## 3. Results

Mean age of patients with septic shock is 57.32 ± 8.71 years, patients with sepsis 61.00 ± 9.25 years, whereas mean age of healthy control individuals is 59.11 ± 9.64 years. The mortality rate was 26% and 35.13% in sepsis and septic shock group, respectively. No statistically significant difference has been found between septic shock, sepsis, and control groups in terms of age and sex variables (Table 1). 

**Table 1:** Demographic features of septic shock, sepsis, and control groups.

	Septic shock group	Sepsis group	Control group
Sex, n (%) Female Male Age (years) mean ± SD	21(%33.3) 32(%50.8) 57.32 ± 8.71	23(%36.5) 37(%58.7) 61.00 ± 9.25	22(%34.9) 38(%60.3) 59.11 ± 9.64

The levels of thiols-disulphides and distributions of ratios of reduced thiol, oxidized thiol, and thiol oxidation reduction by groups are given in Table 2. 

**Table 2:** Antioxidant levels of study groups.

	Septic shock groupn: 53	Sepsis groupn: 60	Control groupn: 60	P-value
TTL(µmol/L)** NTL(µmol/L)** DISULPHIDE (µmol/L) RTR** OTR** TORR**	194.94 ± 82.29 121.89 ± 61.70 36.53 ± 17.68 59.12 ± 17.63 20.86 ± 8.34 271.50(14.90/45411.10)*	226.03 ± 78.62 134.75 ± 68.03 45.65 ± 21.50 60.73 ± 14.09 19.65 ± 7.05 327.30(54.20/1192.40)*	417.35 ± 74.21 307.99 ± 62.63 54.68 ± 27.23 74.05 ± 1.66 13.16 ± 5.61 558.20(117.20/15549.60)*	<0.01 <0.01 <0.01 <0.01 <0.01 <0.01

*Values are presented as median (minimum/maximum). **TTL: total thiol level, NTL: native thiol level, RTR: reduced thiol ratio, OTR: oxidized thiol level, and TORR: thiol oxidation reduction ratio.

All researched parameters (total thiol level, native thiol level, disulphide level, reduced thiol level, oxidized thiol ratio, and thiol oxidation reduction ratio) were significantly lower in patients with sepsis and septic shock than control group (P < 0.01). There was no significant difference between the septic shock and sepsis group.

The levels of disulphides, total thiols, and native thiols and distributions of ratios of reduced thiol, oxidized thiol, and thiol oxidation reduction between survivors and non survivors of patients are given in Table 3. Reduced thiol ratio and thiol oxidation reduction ratio are significantly lower in patients’ survivor group (P < 0.05). 

**Table 3:** Levels of antioxidant molecules investigated between survivors and nonsurvivor groups (including septic shock).

	Survivorsn: 39	Nonsurvivorsn: 74	P	95 % CI
TTL(µmol/L)** NTL(µmol/L)** DISULPHIDE (µmol/L) RTR** OTR** TORR**	201.84 ± 73.44 115.67 ± 53.73 43.12 ± 15.30 55.33 ± 12.36 22.33 ± 6.17 231.20(14.90/45411.10)*	209.80 ± 85.17 130.04 ± 66.56 39.88 ± 22.19 61.68 ± 16.75 19.16 ± 8.37 333.95(54.20/679.60)*	0.622 0.247 0.365 0.040 0.039 0.013	–23.94 to 39.85 –10.11 to 38.86 –10.29 to 3.82 0.30 to 12.37 –6.18 to –0.16 –19.99 to 1562.39

*Values are presented as median (minimum/maximum). **TTL: total thiol level, NTL: native thiol level, RTR: reduced thiol ratio, OTR: oxidized thiol level, and TORR: thiol oxidation reduction ratio.

Distribution of values of erythrocyte sedimentation rate, C-reactive protein, and procalcitonin between survivors and nonsurvivors are given in Table 4. All researched parameters (erythrocyte sedimentation rate, C-reactive protein, and procalcitonin) are significantly lower (P < 0.01) in patients in survivor group compared to the nonsurvivor group.

**Table 4:** Acute phase reactants of survivors and nonsurvivors in the sepsis group (including septic shock).

	Survivors n: 39	Nonsurvivors n: 74	P
Erythrocyte sedimentation rate (ESR, mm/h) C-reactive protein (CRP, mg/L) Procalcitonin (PCT, ng/mL)	2.84 (1.80/4.40)* 41.50 (9/125)* 6.03 (0.05/93.44)*	50(1/141)* 164.19 (3.22/571.23)* 136.28 (14.50/549.27)*	<0.01 <0.01 <0.01

*Values are presented as median (minimum/maximum).

No significant correlation has been established between thiol/disulphide values and erythrocyte sedimentation rate (ESR), C-reactive protein (CRP), and procalcitonin (PCT).

## 4. Discussion

The aim of this research is to measure the levels of thiol and disulphide in patients with sepsis and septic shock, which is common in intensive care units and examine their relationship with acute phase reactants. The results in this study have been reported as follows: 1) Thiol-disulphide balance is impaired in patients with septic shock and sepsis. 2) Native thiol level, total thiol level, and dynamic disulphide bond level have been found statistically lower in septic shock and sepsis groups. 3) Native thiol level, total thiol level, and dynamic disulphide bond level have been found higher in survivor group compared to nonsurvivor group but not statistically significant. 

In this study it has been found that total thiol level, native thiol level, disulphide value as well as thiol oxidation reduction ratio, are statistically significantly lower in sepsis group compared to control. These results correlate with previous studies investigated in patients with sepsis and show that oxidant and antioxidant balance are deteriorated in patients with sepsis and septic shock [10–12]. However, reduced thiol ratio, oxidized thiol ratio, and thiol oxidation reduction ratio have been found higher than control group. This may be due to low disulphide levels. Therefore, the proportional changes have been found higher in sepsis group than control group. Septic shock is a subset of sepsis associated with mortality in the range of 40%–50% that can be identified by the use of vasopressor therapy and the presence of elevated lactate levels (>2 mmol/L) despite adequate fluid resuscitation [13]. Mortality has been estimated to be ≥10% in patient with sepsis and ≥40% in patients with septic shock [14]. Tissue hypoperfusion and multiple organ failure are more common in septic shock. This suggests that tissue damage increases as a result of more oxidative stress. The low level of total thiol level, native thiol level, and disulphide value shown in septic shock group support this theory.

Sepsis is a disease that can progress to inflammation and multiple organ failure in the endothelial system triggered by an increase in the synthesis of proinflammatory molecules in the pathogenesis of sepsis [12]. There are a few studies on antioxidant molecules in adult patients with sepsis. When the literature has been researched, current study is seen as the first manuscript which evaluates thiol disulphide balance in serum of adult patients with septic shock and sepsis using new, automated, and colorimetric method.

Although it has been shown that thiols play a role in the pathogenesis of many disease, such as ankylosing spondylitis, lung cancer, rheumatoid arthritis, diabetic nephropathy, sickle cell disease, gastric cancer, and juvenile idiopathic arthritis [15–21], few studies have been conducted with respect to sepsis [22,23].

Ayar G et al. measured thiol-disulphide homeostasis as oxidative stress marker in critically ill children with sepsis [23]. They found that the levels of native thiol, total thiol, and disulphide were significantly decreased in patients with septis shock and sepsis. They found no significant difference in terms of reduced thiol ratio, oxidized thiol ratio, and thiol oxidation reduction ratio between healthy and sepsis groups. In addition, it has been found that levels of native thiol, total thiol, and disulphide were higher in nonsurvivor group compared with survivor group. This indicates that there is no correlation between levels of native thiol, total thiol, disulphide, and disease activity in patients with sepsis.

In one study which involves 100 patients with sepsis and 50 healthy individuals, it is reported that levels of oxidants are higher in septic adult patients [24]. Additionally, this study showed that oxidative stress increases the level of antioxidant molecules and a reversed correlation between antioxidant molecules and multi organ failures. In another study managed by Lorente et al, the levels of antioxidant molecules were compared between nonsurvivors and survivors. It has been reported that there is an association between total antioxidant state and mortality in patients with severe sepsis [10]. In this study, it has been shown that decreased antioxidant state increases the severity of sepsis and is associated with mortality. In another study which involved only 17 patients with sepsis, no difference has been identified between survivor and nonsurvivor group patients [3]. In this study, native thiol level, total thiol level, and dynamic disulphide bond level have been found higher in survivor group compared to nonsurvivor group but this increase is not statistically significant. It may be due to insufficient number of patients.

Traditional markers of systemic inflammation, such as CRP, ESR and white blood cell count, have been proven to be of limited utility in such patients due to their poor sensitivity and specifity for infections. In this study, erythrocyte sedimentation rate, C-reactive protein, and procalcitonin were compared between survivors and nonsurvivors. All parameters have been higher in nonsurvivor group. This study showed that traditional markers of systemic inflammation which have low specifity is associated with the severity of sepsis and compatible with literature [25]. However, no significant relationship has been found between thiol levels and systemic inflammation biomarkers. This suggests that thiol levels play a role in the pathogenesis of sepsis but cannot be used to follow disease severity.

In this study, thiol/disulphide homeostasis has been studied in adult critical care patients with sepsis and septic shock by a novel method and found that thiol levels are less than control patients compared to patients with sepsis and septic shock.

Limited number of patients, not using another antioxidant parameter for comparison, and being single centred are limitations of this study. Neurological signs such as consciousness and delirium, diversity of critical ill patients, insufficient medical history, and no information about the drugs used are important factors that may affect the results.

In conclusion, this research found out that dynamic thiol/disulphide balance is impaired in septic shock and sepsis. This antioxidant regulation can be the underlying cause of many serious conditions or result of pathogenesis in critically ill adult patients. Using this novel method will make detecting antioxidant molecules easy but should not be used to screen the disease activity.

## Acknowledgements

No support has been received from any funding organization for this research.

## Informed Consent

Approval of the Ethics Committee of Gaziantep University Faculty of Medicine has been obtained for this study (26/01/2018-2018/48). Particular data regarding medical record, age, and sex have been obtained for each participant.
